# Editorial for the Special Issue on Wearable and Implantable Bio-MEMS Devices and Applications

**DOI:** 10.3390/mi15080955

**Published:** 2024-07-26

**Authors:** Bowen Ji, Kunpeng Gao

**Affiliations:** 1 Unmanned System Research Institute, Northwestern Polytechnical University, Xi’an 710072, China; 2 National Key Laboratory of Unmanned Aerial Vehicle Technology, Integrated Research and Development Platform of Unmanned Aerial Vehicle Technology, Northwestern Polytechnical University, Xi’an 710072, China; 3 Ministry of Education Key Laboratory of Micro and Nano Systems for Aerospace, School of Mechanical Engineering, Northwestern Polytechnical University, Xi’an 710072, China; 4 College of Information Science and Technology, Donghua University, Shanghai 201620, China

Wearable and implantable bio-MEMS sensors and actuators have attracted tremendous attention in the fields of health monitoring, disease treatment, and human–machine interaction, to name but a few [1,2,3,4,5,6]. A variety of devices have been developed in the last decade covering different aspects of advanced materials [7,8,9,10,11,12], device designs [13,14,15,16,17], manufacturing processes [18,19,20,21,22], and packaging methods [23,24,25,26,27], as well as system integration [28,29,30,31,32]. During our preparation of this Special Issue, we had the following questions in mind: (1) What is the most important concern for researchers in engineering and scientific fields, and what are the differences between their concerns? (2) What is the current state of the art for wearable/implantable devices and systems? 

In this Special Issue, we focus on the latest advancements, current challenges, and new opportunities in the area of bio- and medical MEMS devices and applications. Optimized design methods, micromachining technology, and microsystem integration for any potential application are covered in this issue. The goal of this Special Issue is to stimulate the academic community’s awareness of the great promise that novel wearable and implantable devices and systems hold for improving the quality of people’s lives.

Out of the six articles published in this Special Issue volume, five are original research papers and one is a review article. All six papers were submitted from China, with different aspects of concern: including the use of a perforated structure to improve the sensitivity of MEMS devices for respiratory monitoring; manufacturing methods for localized surface hydrophilicity tailoring; classification algorithms for EEG signal detection and hand gesture recognition; and applications using MEMS devices for detecting sEMG signals and monitoring biomarkers of chronic disease.

(1) Novel design: Cao et al. [33] from Peking University propose a perforated temperature sensor that can be worn below the nasal cavity to monitor breath. The sensing system consists of two perforated temperature sensors, signal conditioning circuits, a transmission module, and a supporting analysis algorithm. Most importantly, these authors found that this novel perforated structure effectively enhanced the sensitivity of the system to 1.4‰ °C^−1^ and shortened the response time to 0.07 s.

(2) Novel fabrication: Ji et al. [34] from Northwestern Polytechnical University used an atmospheric-pressure Ar/H_2_O microplasma jet with a nozzle diameter of 100 μm to site-selectively tune the surface hydrophilicity of a polyimide (PI) film. As we know, PI is widely used as the flexible substate for wearable and implantable MEMS devices; however, its poor hydrophilicity limits its potential for applications in flexible electronics. In this study, the wettability of the PI surface was significantly enhanced after microplasma modification, and the WCA could be adjusted by varying the applied voltage, water vapor content, plasma treatment time, and storage time.

(3) Novel algorithms: First, Wang et al. [35] from Donghua University propose an algorithm for classifying EEG signals based on canonical correlation analysis (CCA) and integrated with adaptive filtering. This algorithm can enhance the detection of steady-state visual evoked potentials (SSVEPs) in a brain–computer interface (BCI) speller, and is more suitable for wearable environments where high-density EEG signals are not easy to collect. Second, Wang et al. [36] from the Dalian Neusoft University of Information investigate static and dynamic gesture-recognition methods based on miniature inertial sensors. In their study, hand-gesture data were obtained through a data glove and preprocessed using Butterworth low-pass filtering and normalization algorithms. Their experimental results demonstrate that the random forest algorithm achieved the highest recognition accuracy and shortest recognition time for static gestures, and the addition of the attention mechanism significantly improved the recognition accuracy of the LSTM model for dynamic gestures, with a prediction accuracy of 98.3%.

(4) Novel system and application: First, Liang et al. [37] from Northwestern Polytechnical University propose a novel wrist-worn system with four sEMG acquisition channels and a high common-mode rejection ratio (CMRR), greater than 120 dB. This system is fabricated using flexible circuit technologies and is encapsulated in a soft skin-friendly silicone gel, finding potential applications in natural and intuitive human–computer interaction and physiological state monitoring. Second, Yuan et al. [38], also from Northwestern Polytechnical University, summarize the biomarkers in interstitial fluid, introduce and explain the extraction methods for interstitial fluid, and discuss the application of epidermal wearable biosensors for the continuous monitoring of markers in clinical biology. In addition, the current needs, development prospects, and challenges in this area of wearable-device applications are briefly discussed.

In summary, we hope that this Special Issue on wearable and implantable bio-MEMS devices and applications will offer readers a good overview of the current state of the art in this fast-growing area of research, as well as an introduction to some of the newest techniques developed in this field.
